# Impact of a ground intermediate transport from the helicopter landing site at a hospital on transport duration and patient safety

**DOI:** 10.1186/s13049-023-01124-7

**Published:** 2023-10-24

**Authors:** Dominik J. Hoechter, Bjarne Schmalbach, Merve Schmidt, Stephan Prueckner, Andreas Bayer

**Affiliations:** 1 Department of Anesthesiology, LMU Klinikum, University Hospital, Ludwig- Maximilians-University Munich, Marchioninistr. 15, D-81377 Munich, Germany; 2 German Air Rescue Service Association “DRF Luftrettung”, air base Munich, Elisabeth-Stöber-Straße 71, D-81377 Munich, Germany; 3 Scientific research department, German Air Rescue Service Association “DRF Luftrettung”, Rita-Maiburg-Straße 2, D-70794 Filderstadt, Germany; 4 German Air Rescue Service Association “DRF Luftrettung”, air base Niebuell Gather Landstraße 75, D-25899 Niebuell, Germany; 5 Institute of Emergency Medicine and Management in Medicine, LMU Klinikum, University Hospital, Ludwig-Maximilians-University Munich, Schillerstr. 53, D-80336 Munich, Germany

**Keywords:** HEMS, Helicopter emergency services, Intermediate ground transport, Transportation duration, Remote helicopter landing site

## Abstract

**Background:**

Helicopter emergency medical service provides timely care and rapid transport of severely injured or critically ill patients. Due to constructional or regulatory provisions at some hospitals, a remote helicopter landing site necessitates an intermediate ground transport to the emergency department by ambulance which might lengthen patient transport time and comprises the risk of disconnection or loss of vascular access lines, breathing tubes or impairment of other relevant equipment during the loading processes. The aim of this study was to evaluate if a ground intermediate transport at the hospital site prolonged patient transport times and operating times or increases complication rates.

**Methods:**

A retrospective analysis of all missions of a German air rescue service between 2012 and 2020 was conducted. Need of a ground transport at the accepting hospital, transfer time from the helipad to the hospital, overall patient transport time from the emergency location or the referring hospital to the accepting hospital and duration of the mission were analyzed. Several possible confounders such as type of mission, mechanical ventilation of the patient, use of syringe infusion pumps (SIPs), day- or nighttime were considered.

**Results:**

Of a total of 179,003 missions (92,773 (51,8%) primary rescue missions, 10,001 (5,6%) polytrauma patients) 86,230 (48,2%) secondary transfers) an intermediate transport by ambulance occurred in 40,459 (22,6%) cases. While transfer times were prolonged from 6.3 to 8.8 min for primary rescue cases (*p* < 0.001) and from 9.2 to 13.5 min for interhospital retrieval missions (*p* < 0.001), the overall patient transport time was 14.8 versus 15.8 min (*p* < 0.001) in primary rescue and 23.5 versus 26.8 min (*p* < 0.001) in interhospital transfer. Linear regression analysis revealed a mean time difference of 3.91 min for mechanical ventilation of a patient (*p* < 0.001), 7.06 min for the use of SIPs (*p* < 0.001) and 2.73 min for an intermediate ambulance transfer (*p* < 0.001). There was no relevant difference of complication rates seen.

**Conclusions:**

An intermediate ground transport from a remote helicopter landing site to the emergency department by ambulance at the receiving hospital had a minor impact on transportation times and complication rates.

**Supplementary Information:**

The online version contains supplementary material available at 10.1186/s13049-023-01124-7.

## Background

Time to initiation of advanced care is of essence for acutely injured or critically ill patients and might directly reflect in outcomes [1]. In addition to providing advanced prehospital patient care on scene, helicopter emergency medical service (HEMS) facilitates rapid transport to high care facilities [2–4]. It therefore plays a pivotal role in both prehospital emergency care and timely interhospital transfer of critically ill patients.

In order to ensure a swift transfer from the helicopter to the emergency department or treatment unit, in Germany, several guidelines and regulations of the care of acutely injured or poly-traumatized patients outline the localization of helicopter landing sites at hospitals: The German Association of Trauma Surgery defines a helicopter landing site “in close proximity of the emergency room or resuscitation bay” as a prerequisite for a regional trauma center [5]. Likewise, the German Social Accident Insurance (IAG) lists a constantly operational helicopter landing site near the emergency department or resuscitation bay as a criterion for hospitals that participate in the care of severely injured patients [6]. The directive of the Federal Joint Committee (G-BA) - which is the highest decision-making body of the joint self-government of physicians, dentists, hospitals and health insurance funds in Germany and issues directives for the benefit catalogue of the statutory health insurance funds - requests a helicopter landing site at an advanced emergency care providing hospital that allows for air-bound patient transfer without an intermediate transport on the ground [7].

However, constructional or regulatory provisions – by air traffic regulations or noise pollution control acts - sometimes do not allow for the installation of a helicopter landing site or public interest site right next to the emergency department. Hence, an intermediate transport from the helicopter landing site to the emergency department by ambulance might be necessary which might lengthen the overall transport time and comprises the risk of line or tube disconnection during the loading processes.

The aim of this study was to evaluate if a ground intermediate transport at the hospital site prolonged patient transport times and mission times or increased complication rates.

## Methods

A retrospective analysis of the German air rescue service DRF Luftrettung (DRF Luftrettung gAG, Filderstadt, Germany) database was conducted. The database collects all operational data of the 29 DRF helicopters in Germany. The helicopters are alerted as part of emergency medical services as well as the interhospital retrieval network. The medical crew on the helicopters consists of a pre-hospital emergency physician (mostly specialists in anesthesiology, surgery, or internal medicine) and a HEMS-TC (helicopter emergency medical system technical crew member) moreover qualified as a paramedic. Each mission is documented in a standardized online-database (HEMSDER-Database, Convexis, Germany). The collected data included flight times as well as medical details on the patient and care.

This retrospective study included all missions of DRF helicopters with a patient transported to a hospital from 2012 to 2020. The following times were collected: time from landing at the hospital site to handover in the hospital (transfer time), time from helicopter start at the emergency location or the referring hospital to handover in the hospital (patient transport time), time from helicopter start at the emergency location or the referring hospital to landing at the accepting hospital (flight time), time from helicopter landing at the emergency location or referring hospital to handover in the hospital (patient contact time) as well as the times from patient contact until operational readiness after patient handover (mission time) (Table 1). The following patient characteristics were collected: age, diagnosis, intubated/ventilated, need for vasopressors, use of syringe infusion pumps (SIPs), transport by incubator, transport of patients with a cardiopulmonary assist device (e.g. extracorporeal membrane oxygenation (ECMO) or (percutaneous) ventricular assist devices).

**Table 1:** Description of the time intervals that were examined

Time interval	Description
Transfer time	time from landing at the hospital site to handover in the hospital
patient transport time	time from helicopter start at the emergency location or the referring hospital to handover in the hospital
patient contact time	time from helicopter landing at the emergency location or referring hospital to handover in the hospital
mission time	time from patient contact until operational readiness after patient handover

As urgency likely differed between primary rescue missions and secondary hospital transfers, missions were categorized in primary rescue missions and secondary interhospital patient transfer missions.

Several secondary analyses were performed to depict the many-sidedness of HEMS: Since the above-mentioned provisions mainly apply for the care of multiple injured or trauma patients, a subgroup-analysis was performed segregating primary rescue missions in primary rescue missions for polytraumatized patients and primary rescue missions for all other emergencies.

In addition, missions were categorized by day or nighttime and by season; here, spring and autumn were combined to “mid-season” due to similar meteorological conditions including capricious temperatures, rainfall as well as windy or foggy conditions; further, the impact of transports by means of incubators and transports with a paracorporeal support device (extracorporeal membrane oxygenation (ECMO), intraaortic balloon pump (IABP), percutaneous mechanical support (e g Impella®)) was explored.

Moreover, information on the helicopter type was gathered where possible to account for differences resulting from diverging helicopter configurations.

For the descriptive analysis of numerical variables, the mean and standard deviation were computed, for categorial and dichotomous variables the frequency and proportion in percent were calculated. Chi-squared-test was used for comparative analysis of frequency distributions and t-tests, or ANOVA were calculated for metric variables. Effect sizes were calculated using Cramer’s V and R-squared or Cohen’s d respectively.

A p-value of 0.05 or less was considered statistically significant. Analyses were calculated using programming language R and the package *effectsize* [8].

## Results

**Fig. 1:**
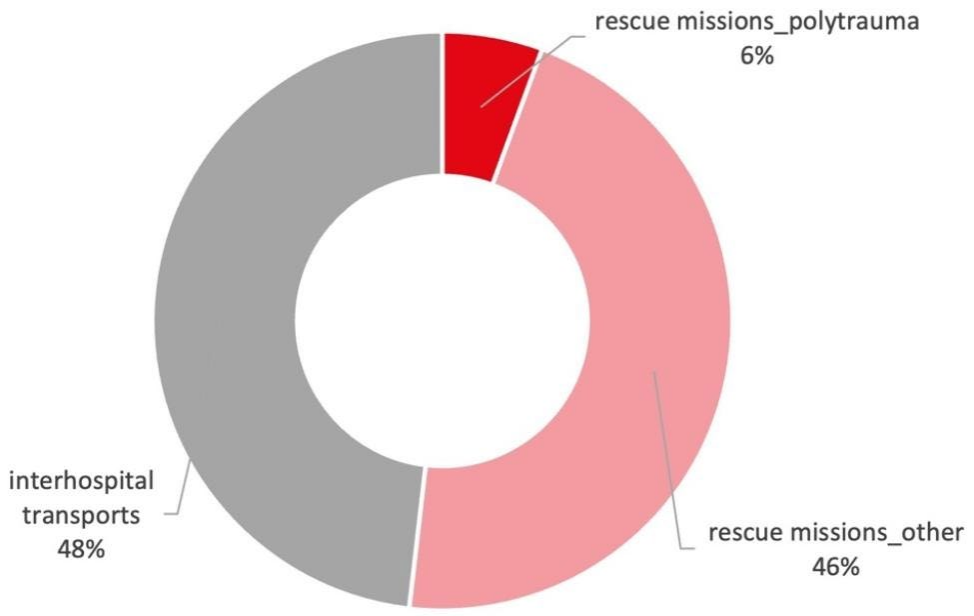
Number of transports of a HEMS association during a nine-year period

From 2012 to 2020 DRF helicopters realized 179,003 patient transports. 92,773 (51,8%) were primary rescue missions of which 10,001 (10,8% of primary rescue, 5,6% of all transports) cases the main diagnosis was polytrauma, while 82,772 (89,2% of primary rescue, 46,2% of all transports) were due to other emergencies comprising medical, neurological, or single site traumatic injuries. 86,230 (48,2%) were secondary transfers from one hospital to another. An intermediate transport by ambulance from the landing site to the hospital occurred in 40,459 (22,6% of all transports) cases (Fig. 1).

Demographics and severity of illness or injury expressed via the National Advisory Committee for Aeronautics (NACA) score differed between the groups of emergency patients (primary rescue) versus retrieval patients (interhospital transport) and are depicted in Table 2 (Table 2). Two thirds of the patients were male with slightly more male patients among the emergency patients. Compared to patients of primary rescue missions with an average of 48.0 years, retrieval patients were markedly older with an average age of 58.0 years (Table 2).

**Table 2:** Demographics and medical device use: age and sex of patients, NACA score and number of intubated and ventilated patients as well as requirement of catecholamine therapy or syringe infusion pump use. Age in years: mean and standard deviation; all other: number and percent of the respective cohort

	rescue mission		interhospital transport	
	n = 92,773		n = 86,230	
age (a)	48.0	± 25.0	58.0	± 23.0
female (n/%)	30,955	(34.3%)	30,647	(36.7%)
NACA 3 (n/%)	34,859	(41%)	12,010	(14%)
NACA 4 (n/%)	21,179	(25%)	27,801	(33%)
NACA 5 (n/%)	25,904	(30%)	43,542	(51%)
NACA 6 (n/%)	1,999	(2.4%)	1,310	(1.5%)
intubation (n/%)	15,259	(16.4%)	20,844	(24.2%)
mechanical ventilation (n/%)	16,241	(17.5%)	26,496	(30.7%)
catecholamines (n/%)	6,319	(6.8%)	18,890	(21.9%)
syringe infusion pump (n/%)	1,441	(1.6%)	28,008	(32.5%)

Among emergency patients, traumatic single injury, intracerebral hemorrhage, other neurological emergencies like stroke and cardiovascular emergencies were the leading diagnoses. With regards to the severity of the emergency, 41% of these patients were assigned to NACA score 3, 25% to NACA 4 and 30% to NACA score of 5. Intubation was required in 16.4% of the emergency patients and ventilation in 17.5%. Catecholamines were applied on 6.8% with syringe infusion pump use in only 1.6%.

In interhospital transfer, main diagnoses included stroke, acute coronary syndrome, vascular emergencies like aortic dissections and neurosurgical pathologies such as intracranial bleeds and acute respiratory distress syndrome and sepsis. 51% of these patients were rated with an NACA score of 5. 23,5% of patients were intubated and 30,0% were dependent on a ventilator. 21,5% received catecholamine therapy and in 32,1% of the cases syringe infusion pumps were utilized (Table 2).

An intermediate ground transport by ambulance occurred in 20.6% of emergency patients and 24.7% of interhospital transfers.

Transfer times from the helicopter landing site to the emergency department were 6.3 and 9.2 min for primary rescue and interhospital transfer and prolonged to 8.78 and 13.5 min respectively when a ground intermediate transport took place (*p* < 0.001; *d* = 0.34 and 0.38).

Patient transport time without and with intermediate transport was 14.8 versus 15.8 min (*p* < 0.001, *d* = 0.1) in primary rescue and 23.5 versus 26.8 min (*p* < 0.001, *d* = 0.16) for interhospital transfer. Details for flight time and patient contact time are listed in Table 3 (Table 3). Linear regression analysis of selected determinants on patient transport time revealed a mean time difference of 3.91 min for mechanical ventilation of a patient (*p* < 0.001), 7.06 min for the use of SIPs (*p* < 0.001) and 2.73 min for an intermediate ambulance transfer (*p* < 0.001); in primary rescue missions, the difference was 1.69 min for ventilation, 4.52 for use of SIPs and 1.03 min for an intermediate transfer.

**Table 3:** Transfer and transportation times: please refer to Table 1 for definitions of time intervals examined

	rescue mission				interhospital transport			
	intermediate ground transport				intermediate ground transport			
	w/o	w	p-value	Cohens’d	w/o	w	p-value	Cohens’d
transfer time	6.27	8.78	< 0.001	0.34	9.16	13.48	< 0.001	0.38
patient transport time	14.79	15.81	< 0.001	0.10	23.51	26.77	< 0.001	0.16
patient contact time	44.10	47.11	< 0.001	0.20	65.47	72.74	< 0.001	0.24
mission time	74.23	78.03	< 0.001	0.13	109.6	120.2	< 0.001	0.21

Mission time – defined from landing at the emergency site or the referring hospital until operational readiness – was 74.2 min versus 78.0 min (*p* < 0.001, *d* = 0.13) in primary rescue and 109.6 versus 120.2 min (*p* < 0.001, *d* = 0.21) for interhospital transfer cases.

Overall, complications were documented in 1,184 of 138,544 (0,9%) cases in the group without intermediate ambulance transport versus 441 of 40,459 (1,1%) cases in the intermediate transport group (*p* < 0.001; *V* = 0.01). Similarly, complication rates were 0.5%, and 0.6% (*p* = 0.051; *V* < 0.01) in primary rescue and 1.3% and 1.6% (*p* = 0.007; *V* < 0.01) in interhospital transfer. Complications were reported in the categories of medical treatment complication (airway issues, respiratory and hemodynamic complications, iatrogenic injuries), medical equipment failure or organizational issues.

**Fig. 2:**
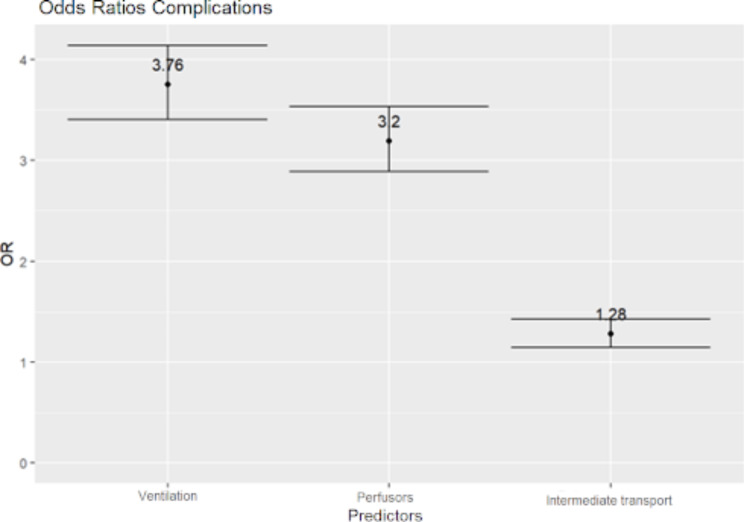
Odds ratio for complications over all mission types for mechanical ventilation, use of syringe infusion pumps and intermediate transport: odds ratio and confidence interval

When assessing the impact of ventilation, use of SIPs and intermediate ground transport on complication rates, the need for ventilation was associated with an odds ratio of 3.76 (3.41–4.15) and use of SIP with an OR of 3.20 (2.89–3.53) while intermediate ground transport resulted in an OR of 1.28 (1.15–1.43) (Fig. 2).

Analysis of variance showed that helicopter model may account for differences in patient transport time (*F*(3,121859) = 4,287, *p* < 0.001, *R*^*2*^ = 0.119) but does not alter transfer time at the hospital site (*F*(3,120151) = 352, *p* < 0.001, *R*^*2*^ = 0.008) (Appendix 1, Supplementary Material 1). Besides, analysis of variance of neither daytime nor season showed significant results of an influence of intermediate transport on the respective times or complication rates. Similarly, an intermediate transport showed no significant effect on times in transports by means of an incubator or in transports of patients with a percutaneous heart or lung assist device. More detailed results in this regard as well as a subgroup analysis of primary rescue missions for polytraumatized patients versus all other emergencies are presented in the Appendix 2 (Tables 1 and 2, Supplementary Material 2).

## Discussion

The study at hand evaluated the effect of an intermediate ground transport by ambulance from the helicopter landing site at a hospital on patient transport times and safety. The analysis was presented for primary rescue missions as well as secondary interhospital retrieval missions since these tend to differ in urgency but also in complexity of patient care and equipment; further, the handover for interhospital retrieval patients might not take place in the emergency department but rather in intensive care units or in theatres and therefore include a longer distance within the hospital. While an intermediate ground transport from a remote landing site by ambulance implies an additional loading on and off process and an additional ground transportation leg, a direct transfer from the helicopter might either involve the use of an elevator in case of roof top landing pads or a walk by foot from a landing site in safe distance from the hospital building to the emergency department.

In this study, an intermediate transport did prolong the transfer time of patients from helicopter landing at the hospital to the handover in the emergency department or in the ICU for emergency or retrieval patients respectively by 2.51 min in primary rescue missions and 4.31 min in secondary interhospital transfers on average. This effect – yet weak in effect – further diminished when assessing the overall patient transport time and there was no relevant impact on the rate of documented complications seen.

HEMS has been established to provide emergency care in otherwise in-accessible locations and a timely hospital admission for critically injured patients [9]. In the past, numerous studies have evaluated the effects of HEMS compared to ground-based emergency services and weighed the benefits of a more intense and invasive treatment and faster transport by HEMS against higher expenses [4, 10, 11]. However, there is scarce investigation on the transfer time from the helicopter landing site to the hospital. Zanic and colleagues report a mean heliport-to-hospital time of seven minutes in Croatian emergency air transport for acute chest pain patients which is akin to our findings [12]. Furthermore, only one published study was identified addressing a transport delay resulting from a remote helicopter landing site [13]. Lerner and colleagues determined a time delay in emergency department arrival of trauma patients resulting from a remote helipad requiring an ambulance transport at a trauma center of 5.2 ± 2.3 min by simply taking the time difference between landing of the helicopter and the arrival at the emergency department but did not compare to transports without intermediate transport. In contrast, this study at hand compared the actual transfer times of ground transport by ambulance (from a remote helipad) to ground transport by foot (from an adjacent helipad) therefor depicting a more realistic comparison. It revealed an effective delay of as few as 2.5 min for emergency patients. While for some patients who are in extremis, any time saving is crucial, the reported delay appears short against a background of a total patient transport time of 16 min and a total prehospital patient contact time of about an hour. Results of the linear regression analysis underlined this trend by displaying the influence of determinants on total patient transport time: mechanical ventilation of a patient or the use of SIPs both exhibited a longer mean differential time span compared to SIPs (5.69; t-value − 17.2) an intermediate ambulance transfer.

The number of reported complications was low at 1% in our cohort, missions during nighttime presented with slightly higher complication rates of 1.3% and the rate of documented complications increased up to 3% in missions with extracorporeal support. While the effect of an intermediate transport on the complication rate was significant, its effect size was minimal (Cramer’s V 0.01). As very likely only complications with clinical relevance were documented, there might be a risk of underreporting critical incidents. While a recent meta-analysis on interhospital transport of critically ill patients comprising 14,969 transports found a pooled rate of adverse events in interhospital transport of 11%, the rate of complications was shown to be as low as 1% when specialized teams performed the retrieval [14, 15].

In contrast to deductions in the past that an additional ground transport leg from the remote helipad would lead to an inherent risk for the patient in form of jostling during the loading processes or dislodgements of tubes and lines, the number of relevant complications did not distinctly differ between missions with or without ambulance ground transport in this study [16]. Moreover, the need of mechanical ventilation or use of SIPs seemed to have a markedly higher impact on complication rates compared to an intermediate transport at the hospital.

As external circumstances might influence performance and framework conditions, we also took daylight and weather conditions into consideration: With the majority of HEMS sites only operating during daylight, 86% of the missions were performed by day and 11% during nighttime. Even though there was a tendency of a higher likelihood of complications for nocturnal missions (1.4% versus 1.0%) and missions with percutaneous cardiopulmonary assist devices (3.0% versus 0.9%), the occurrence of an intermediate transfer did neither have an impact on complication rates nor transfer times.

In addition, neither different helicopter models resulting in disparate patient stretcher handling mechanisms nor seasonal meteorological effects showed a signal to influence the time delay due to intermediate ground transport or complication rates.

Albeit these results question a negative effect of an intermediate transport by ambulance, it should not be left unmentioned that the helicopter landing site probably should be within a certain proximity to the emergency department: in this cohort, in the intermediate transport group, the transfer time from the helicopter landing site to the emergency department was about 9 min on average in primary rescue missions and 13 min in secondary interhospital transports with the majority (75%) of intermediate transports taking no longer than 12 and 17 min respectively.

This study has several limitations of which many are inherent to the retrospective design. Data quality relied on the accuracy of the documentation during the missions. The documentation of complications didn’t allow to determine their nature or whether complications occurred during air or ground transport. Further, there are scarce data on incubator missions or transports on paracorporeal devices as these are often performed with a dedicated team using a separate documentation. Unfortunately, attempts to retrieve these data by deduction from other information such as hospital sites and their helicopter landing facilities were unsuccessful. Hence, the results of the corresponding analysis should be interpreted cautiously.

The missing link to clinical patient outcome – as documentation of the transport ended with handover in the hospital – presents a major shortcoming of this study.

The large sample size provided a comprehensive picture of helicopter transports and provided a robust data fundament. However, this entails the risk that statistical significance is easily reached due to the high number of observations rather than the magnitude of the effect. Effect sizes were calculated and provided to account for here.

## Conclusions

In conclusion, an intermediate transport from a remote landing site at the hospital did prolong the transfer time of patients from the helicopter landing to the handover in the emergency department or in the ICU for emergency or retrieval patients by few minutes. This effect – yet weak in effect – further diminished when assessing the overall patient transport time. An impact on the rate of documented complications was of subordinated effect.

### Electronic supplementary material

Below is the link to the electronic supplementary material.

Supplementary Material 1

Supplementary Material 2

## Data Availability

The data that support the findings of this study are available from German Air Rescue Service Association “DRF Luftrettung” but restrictions apply to the availability of these data, which were used under license for the current study, and so are not publicly available. Data are however available from the authors upon reasonable request and with permission of German Air Rescue Service Association “DRF Luftrettung”.

## Abbreviations

DRF: German air rescue service DRF Luftrettung (DRF, Filderstadt, Germany)
G-BA: Federal Joint Committee
HEMS: Helicopter emergency medical service
HEMS-TC: Helicopter emergency medical system technical crew member
IAG: German Social Accident Insurance
ICU: Intensive care unit
NACA: National Advisory Committee for Aeronautics
SIP: Syringe infusion pump
