# Supplementary material for: Impact of a ground intermediate transport from the helicopter landing site at a hospital on transport duration and patient safety
Source: Scand J Trauma Resusc Emerg Med. 2023 Oct 24;31:58. doi: 10.1186/s13049-023-01124-7 (PMC10598984; doi:10.1186/s13049-023-01124-7)
Supplement: Supplementary file 1 — Supplementary Material 1 [file 13049_2023_1124_MOESM1_ESM.docx]

Appendix 1: Helicopter type, daytime, season, transport by incubator or with extracorporeal device

The helicopter types in use were the following: Aérospatiale Alouette II, models 206, 212 and 412 by Bell, Bo105 by Messerschmitt-Boelkow-Blohm, BK 117B-2 and BK 117C-1 (Messerschmitt-Boelkow-Blohm/ Kawasaki Heavy Industries) as well as EC135 and H135 and EC145 and H145 by Eurocopter/ Airbus. As some of the types were used as seldom as less than ten times or were taken off the aviation market many years ago, analysis was limited to the four most frequently and recently used models EC135 (60,640 missions), EC145 (28,201 missions), H135 (3,865 missions) and H145 (36,400 missions). The proportion of missions with intermediate transport at the hospital site were 28% in EC135, 18% in EC145, 13% in H135 and 20% in H145. Complication rates ranged between 0.7% and 1.3% depending on helicopter model and intermediate transport (*p*=0.001). Analysis of variance showed that helicopter model may account for differences in patient transport time (*F*(3,121859)=4,287, *p*<0.001, *R^2^*=0.119) but not in regard to transfer time at the hospital site (*F*(3,120151)=352, *p*<0.001, *R^2^*=0.008).

154477 (86%) of the missions were performed during daylight and 20226 (11%) during nighttime. Analysis of variance for day or nighttime missions and intermediate transport on the respective durations showed significant results but all *R^2^* values were below 0.05, indicating at most a minimal influence. While there was an overall tendency towards a higher rate of complication during nighttime (1.3%) compared to daytime (0.6%), there was no difference seen when stratified by intermediate transport (*p*=0.78).

The distribution of the missions according to season were as follows: 76884 (43%) in summer, 55056 (31%) in winter and 47060 (26%) in mid seasons. As before, analysis of variance for day or nighttime missions and intermediate transport on the respective durations showed significant results but all *R^2^* values were below 0.05. Complication rates did not differ (*p*=0.78).

Among the 179,003 missions in the database, 2367 (1.3%) transports were identified as transport by means of an incubator of which only ten comprised an intermediate ground transport according to the database. Here as well, analysis of variance on the respective durations showed significant results but again all *R^2^* values were below 0.05, indicating at most a minimal influence.

Further, 1686 (0.9%) transports of patients with a (percutaneous) heart or lung assist device were documented in the database. 405 were reported being carried out with an intermediate transport. Here as well, analysis of variance on the respective durations showed significant results but again all *R^2^* values were below 0.07. Complication rates were markedly higher (3%) in transports with assist devices compared to other transports (0,9%), however there was no difference among the assist device transports when categorized by intermediate transport (3.0% versus 3.2%, *p*=0.78).
