# Supplementary material for: Impact of a ground intermediate transport from the helicopter landing site at a hospital on transport duration and patient safety
Source: Scand J Trauma Resusc Emerg Med. 2023 Oct 24;31:58. doi: 10.1186/s13049-023-01124-7 (PMC10598984; doi:10.1186/s13049-023-01124-7)
Supplement: Supplementary file 2 — Supplementary Material 2 [file 13049_2023_1124_MOESM2_ESM.docx]

Appendix 2:

Table A1: subgroup analysis of primary rescue missions: polytrauma patients versus all other emergency patients: demographics and medical device use: age and sex of patients, NACA score and number of intubated and ventilated patients as well as requirement of catecholamine therapy or syringe infusion pump use. Age in years: mean and standard deviation; all other: number and percent of the respective cohort

|  | rescue mission - polytrauma | | rescue mission - other | |
| --- | --- | --- | --- | --- |
|  | n=10001 | | n=82772 | |
|  |  |  |  |  |
| age (a) | 43 | ±20 | 48 | ±25 |
| female (n/%) | 2,504 | (26%) | 28,451 | (35.3%) |
|  |  |  |  |  |
|  |  |  |  |  |
| NACA 3 (n/%) | 0 | (0%) | 34,859 | (46%) |
| NACA 4 (n/%) | 0 | (0%) | 21,179 | (28%) |
| NACA 5 (n/%) | 9693 | (97%) | 16,211 | (21%) |
| NACA 6 (n/%) | 291 | (2.9%) | 1,708 | (2.3%) |
|  |  |  |  |  |
|  |  |  |  |  |
| intubation (n/%) | 4,961 | (49.6%) | 10,298 | (12.4%) |
| mechanical ventilation (n/%) | 5,162 | (51.6%) | 11,079 | (13.4%) |
|  |  |  |  |  |
|  |  |  |  |  |
| catecholamines (n/%) | 1,689 | (16.9%) | 4,630 | (5.6%) |
| syringe infusion pump (n/%) | 438 | (4.4%) | 1,003 | (1.2%) |

Among the group of primary rescue patients, 10,001 were polytraumatized.

Emergency patients with polytraumatic injury presented in acute danger: over 97 percent of these patients were classified to a NACA score of 5. Approximately half of the patients required intubation and controlled ventilation at the site of accident and in 17% of the patients, catecholamines were necessitated. The injury patterns mainly involved trauma to the head and neck, thoracic and abdominal trauma as well as pelvis and lower extremities.

In the cohort of non-polytrauma primary rescues, intubation and ventilation were applied in 12,4% and 13,4% respectively. Catecholamines were applied in 5,6% of patients.

Table A2: subgroup analysis of primary rescue missions: polytrauma patients versus all other emergency patients: transfer and transportation times:

|  | rescue mission - polytrauma | | | | | rescue mission - other | | | | | | |
| --- | --- | --- | --- | --- | --- | --- | --- | --- | --- | --- | --- | --- |
|  | intermediate ground transport | | |  | intermediate ground transport | | | | |  | |  |
|  | w/o | w | p-value | Cohens'd | w/o | | w | p-value | Cohens'd | |  |  |
| transfer time | 6.33 | 8.50 | <0.001 | 0.30 | 6.26 | | 8.81 | <0.001 | 0.35 | |  |  |
| patient transport time | 15.04 | 15.20 | 0.531 | 0.02 | 14.75 | | 15.88 | <0.001 | 0.11 | |  |  |
| patient contact time | 52.21 | 53.83 | <0.001 | 0.09 | 43.10 | | 46.32 | <0.001 | 0.22 | |  |  |
| mission time | 90.66 | 91.10 | 0.556 | 0.01 | 72.21 | | 76.51 | <0.001 | 0.16 | |  |  |
